# Addiction is Not a Natural Kind

**DOI:** 10.3389/fpsyt.2013.00123

**Published:** 2013-10-04

**Authors:** Jeremy Michael Pober

**Affiliations:** ^1^Department of Philosophy, San Francisco State University, San Francisco, CA, USA

**Keywords:** addiction, natural kinds, theories of addiction, philosophy of neuroscience, philosophy of psychiatry

## Abstract

I argue that addiction is not an appropriate category to support generalizations for the purposes of scientific prediction. That is, addiction is not a natural kind. I discuss the Homeostatic Property Cluster (HPC) theory of kinds, according to which members of a kind share a cluster of properties generated by a common mechanism or set of mechanisms. Leading accounts of addiction in literature fail to offer a mechanism that explains addiction across substances. I discuss popular variants of the disease conception and demonstrate that at least one class of substances that fails to confirm a major prediction of each account. When no mechanism can be found to explain the occurrence of the relevant properties in members of a category, the HPC view suggests that we revise our categories. I discuss options offered by the HPC view, including category revision and category replacement. I then conclude that talk of addiction as a prediction-supporting category should be replaced with categories such as “S-addiction” and “T-addiction,” where S and T are substances or sets of substances of abuse, as these categories are genuine natural kinds.

## Introduction: Addiction and Generalizations

Addiction is discussed as a unified class or condition in philosophical and scientific literature (1). That is, addiction is treated as a category under which substantive generalizations can be subsumed. Basic scientists are concerned with generalizations for the purposes of prediction, clinical scientists for the purpose of diagnosis and treatment of symptoms – ideally on the basis of the knowledge generated by basic science – and philosophers are interested in attributions of responsibility to individuals and to which categories of individuals those attributions apply, as responsibility is an essential component of autonomy^1^. While this tendency to subsume generalizations under the category addiction is prevalent in many fields, it is especially explicit in the dominant paradigm in cognitive neuroscience, the disease paradigm [e.g., Ref. (2, 3)].

But are we justified in making these generalizations? In this discussion, I shall argue that we are not. We ought not treat all cases of addiction as a unified category for scientific, clinical, or philosophical purposes.

### Generalizations and kind terms

Categories in the sciences that support generalizations are referred to as *natural kinds*. To a first approximation, members of a kind share properties in virtue of something that makes them members of this category^2^. Members of a kind share these properties non-accidentally. If the kind is a natural kind, they share these properties in virtue of some scientific basis, be it a shared atomic structure in the physical sciences, or according to the Homeostatic Property Cluster (HPC) view of natural kinds, shared mechanisms in the life sciences.

Many have argued, both in philosophy [e.g., Ref. (4, 5)] and neuroscience [e.g., Ref. (6)] that addiction is a syndrome that varies greatly between individuals on a case by case basis. I agree that there is at least this level of variance, but such variance does not undermine the hypothesis that addiction is a natural kind.

As I’ll discuss, categories or kind terms in the life sciences such as biology and psychology contain a high degree of inherent variation across members, and this variation is not a problem for their ability to count as generalization-subsuming categories. However, this variance among individual members of a kind is unproblematic only insofar as those members share whatever properties they do in virtue of a common mechanism or set of mechanisms.

The dominant paradigm contends that addiction constitutes a natural kind as different expressions of a single *disease*, hence the term (hereafter used) *disease paradigm*. The name suggests that the underlying mechanisms are bio/psychological in nature, thus, according to the disease paradigm, addiction is putatively a natural kind^3^.

My argument is that addiction is not a natural kind because the properties shared by addicted individuals are not explained in virtue of underlying shared mechanisms across substances of addiction.

Because kind term claims are warranted by the existence (or lack thereof) of mechanisms, they are dependent on current science. Thus, my argument more specifically is that the disease conception, as the dominant paradigm in current cognitive neuroscience, fails to provide evidence – and indeed provides disconfirming evidence – that the mechanisms of addiction apply across addictive substances.

Rather, the best suited natural kind terms to explain what we refer to as “addiction” are several subcategories, which, for lack of a better vocabulary, I will distinguish as “S-addiction” “T-addiction,” etc., where S and T stand in for either particular addicting substances or sets of substances.

The argument proceeds as follows. In S 2, I sketch the popular HPC theory of kinds, and in S 3 I suggest how HPC kinds might map onto addiction.

Then, in S 4, I discuss how various models of addiction fail to identify a unified set of mechanisms that explain all cases. I review three mechanistic proposals popular within the disease paradigm. First, I discuss the aberrant learning hypothesis (7), which proposes that habitual behaviors out of the control of conscious/executive systems, realized in dopaminergic projections to and in the dorsal striatum is hypothesized to underlie drug-related behaviors. However, I present evidence that addiction to cannabis does not seem to involve such dopamine activity in the dorsal striatum. Second, I discuss the incentive sensitization hypothesis (8), which proposes that neuroadaptations to the mesolimbic dopamine system cause addicted individuals to consistently overvalue the extent to which they “want” drugs at the expense of other stimuli, including non-drug rewards. However, evidence suggests cannabis addiction seems to involve an increase in the “wanting” of non-drug rewards. Finally, I discuss the frontostriatal dysfunction model [e.g., Ref. (9, 10)]. On this view, multiple circuits realized at least in part in prefrontal cortical areas underlie various aspects of executive control and are inhibited in addiction. However, there is evidence that the neuroadaptations to certain prefrontal areas, including the medial PFC generally but especially the ventromedial and dorsomedial areas, differ between instances of addiction to cocaine and stimulants versus addiction to opiates.

In S 5 I discuss what categories might best explain the phenomena of addiction, and in S 6 I consider the scientific and philosophical implications of the disunity of addiction.

### Terminology and strategy

Before offering an account of natural kinds as well as one of addiction, I should make a note about my strategy in defining those terms. There is no one, uncontested theory of natural kinds, let alone addiction, nor do I wish to settle the dispute about which account is best^4^. I aim to give an account of natural kinds that is generous in what it counts as natural kinds and one of addiction that limits what it counts as addiction to clear cases. For if addiction is not a natural kind given a wide account of natural kinds and a narrow one of addiction, then it stands to reason that the same would be true of a narrower account of kinds – one that demanded more for a category to count as a kind – or a broader one of addiction, where more varied cases count as addiction.

I understand the HPC theory of kinds to be the broadest account available. Other theories allow far less variance within members of a kind, by requiring members of a kind to share either property sets rather than clusters or microstructural essences. In either case, addiction as a natural kind would be a non-starter in virtue of the variance in behaviors that count as addictive from case to case or the lack of a shared essence in life sciences kinds in the first place^5^. Similarly, I restrict my discussion to addiction to substances, rather than addiction to, e.g., gambling.

I should emphasize that this logic only holds for demarcating the *extensions* of addiction and natural kind. That is, it falls short at restricting the possible intensions, i.e., theories explaining in virtue of what the extension “addiction” applies to its instances^6^. And theories within the disease paradigm do not differ on what instances count as addiction so much as they do on how to explain the phenomena exhibited commonly by organisms that count as addicted. I restrict my discussion to theories within the disease paradigm because it is the paradigm investigating addiction at the level most salient for natural kinds, that of mechanisms underlying the property cluster.

## Natural Kinds in Psychology (and Biology)

The HPC theory of natural kinds is designed to balance accounting for the inherent variability in biological and psychological kinds while at the same time preserving explanatory value in terms of prediction and induction (11, 12). This is an attractive model not only because it is gaining traction across the life sciences and being given credence by practicing experimental cognitive neuroscientists [e.g., Ref. (13)], but, more importantly for my purposes, because it is expressly designed to account for the variation inherent to categories such as addiction.

The two basic tenets of the HPC view are: (1) that kinds are defined by a cluster of properties that reliably occur in members of the kind and (2) that the properties co-occur in virtue of some shared causal or mechanistic structure^7^ – the homeostatic in HPC refers to causally maintained co-occurrence. Species are paradigmatic HPC kinds in biology (11, 14).

The challenge for the HPC view is to articulate which categories count as kinds given the fact that members of a kind vary so much. There must be a way to determine: (a) what properties can count in the homeostatic cluster that defines the kind and (b) when a set of properties is truly a cluster and not just an arbitrary set. Both questions are answered by trying to maximize the importance of causal influence: it is properties that are causally basic, as opposed to surface properties that are part of the cluster, and it is when causally basic properties co-occur in virtue of a mechanism or set of mechanisms that the set of properties is truly a cluster (12).

### The HPC view: Key concepts

Properties can be said to be superficial or causally basic: causally basic properties are those by which the other properties can be fully (perhaps reductively) explained. For instance, the causally basic properties of the kind “human” are things like the ability to interbreed and sharing a common ancestry. The superficial properties of the kind human are just those properties that humans share that other categories/kinds don’t: having opposable thumbs isn’t part of the surface property list of humans because it is shared by a higher taxa (primates), but having a complex grammar or a prefrontal cortex larger than other mammals are. The fact that humans have and share these properties can be explained by humans’ shared ancestry and history of breeding.

But these causally basic properties are distinct from the mechanisms that cause them to co-occur in humans. Selection and reproduction might be what cause the surface properties to occur, but the mechanisms of evolution – such as invasive gene suppression and advantageous mutation – are what explain the co-occurrence of those causally basic properties themselves. The mechanisms make the set a cluster, or, better, they make the cluster a *homeostatic* cluster [example from Ref. (12)].

Note that the former causal relationship is stronger than the latter. Uniquely human surface properties can be largely if not entirely explained by the selective history and ancestral genotype of human conspecifics (arguably plus the environmental pressures on them), but no mechanism of gene flow can fully explain those abilities without an additional story being told about the particular historical facts of human reproduction.

Thus, a kind is defined by a cluster of causally basic properties whose clustering is the result of an underlying mechanism/set of mechanisms.

### Delineating kinds from mere categories

Consider an example from evolutionary biology. Squids have eyes much like humans (or whichever taxa our particular kind of visual system belongs to). However, the set of human and squid eyes is considered a homoplasy – a shared trait in virtue of distinct evolutionary histories and mechanisms – rather than an homology, or a shared trait in virtue of a shared ancestral history (14, 15). No putative natural kind to which human eyes belong (e.g., primate eyes, mammal eyes) includes squid eyes (16).

Yet, human and squid eyes have some of the same surface properties (the ability to detect objects, motion, light changes, etc.) and possibly in virtue of the same causally basic properties (a retina, a pupil, etc.). And we can subsume some basic generalizations under a category that includes human and squid eyes, such as that they will both detect an organism of sufficient size moving across their field of view and send this information to the brain. So why don’t human and squid eyes count as a natural kind?

Griffiths refers to the causally basic properties whose co-occurrence is supported not by random chance (as in the case of homoplasy) but by mechanisms to be *maximally* predictive (14). This term is a little misleading in that one might expect maximally predictive categories to be the categories that currently possess the largest cluster of properties.

However, this notion of a maximally predictive category can be made clear by understanding that science is an ever-progressing discipline, and all entities studied by biology or psychology, be they organisms, parts of organisms, mental states, etc., will have properties that have yet to be identified, let alone understood, or explained scientifically. The question, then, is, what is the best way to predict which categories will explain clustering or co-occurrence of properties to be discovered or explained in the future? The answer provided by the HPC view is that the best way to predict future clustering is *not* by present clustering. Rather, the best way to predict future clustering is by reference to mechanisms that explain the co-occurrence of the properties in a cluster.

### HPC kinds and revisability

The fact that natural kind claims are warranted on the basis of mechanisms, rather than on the basis of property clusters, is just one facet of the HPC view’s commitment to the ongoing nature of scientific practice. Wilson and colleagues sum up this commitment well, claiming that “[t]he HPC view carries a commitment to a thoroughgoing naturalism, according to which philosophical reflection on science is continuous with and epistemically regulated by ongoing scientific practice” [Ref. (12), p. 202]. This view entails not only a commitment to categories that will be most useful for predictions in future scientific endeavors, but also a commitment to take seriously the results of those endeavors.

Because science is an ongoing practice, scientific claims are always falsifiable or empirically revisable. This means that all natural kind claims are understood as working hypotheses. In Boyd’s words, they are all *a posteriori* claims (11). More simply, the content of a kind term is whatever the best current science tells us it is: to paraphrase Griffiths (14), addiction means whatever psychology tells us is going on in the mind/brains of addicted individuals.

The upshot of this reasoning is that when I make a claim that “addiction is not a natural kind,” I am making a claim tantamount to a working hypothesis, up for revision if the best current science of addiction changes. But this does not mean the claim is unwarranted *at this time*, it just means it will never be immune from scrutiny in light of new evidence.

### Addiction as HPC kind?

The surface properties of addiction are addictive behaviors or “the addictive phenotype” and the causally basic properties are the person-level functions that putatively explain the phenotype, including psychological factors like habitual desire and loss of control (17), as well as environmental factors captured at a high level of abstraction (e.g., opportunities for employment/advancement). I discuss the causally basic properties proposed by varieties of the disease paradigm in detail in the next section.

Which mechanisms underlie the co-occurrence of these causally basic properties is the primary question addressed in the cognitive neuroscience of addiction. When we discuss “a mechanism for addiction,” what we mean is a mechanism that explains to co-occurrence of some or all of the causally basic properties in the cluster. Thus, according to Everitt and Robbins (7), behavioral control being shifted from prefrontal areas and the ventral striatum to the dorsal striatum is the mechanism responsible for the property of having behavior insensitive to the agent’s conscious desire or executive control, which in turn explains the compulsive drug-taking behavior.

This kind of mechanism, realized in the brain of individuals, is described on a different level – one might say a different kind of mechanism altogether – than the mechanisms of evolution that fix the cluster of properties for the species human. Nonetheless, the important point for kind terms is that they both play the right causal role, that is, they explain the occurrence of the causally basic properties, but to which the causally basic properties cannot be reduced to them.

My argument is that, given the candidate mechanisms proposed in literature, at least one class of cases of addiction as delineated by substance, the proposed mechanism fails to underlie the causally basic properties. I contend, in essence, that the category “addiction” is like a category of eyes that includes both human and squid eyes, whereas the categories “S-addiction” and “T-addiction,” etc., are like categories that include only human eyes (or some other category of eyes defined by homology and not homoplasy).

I should clarify that I am not claiming instances of addiction to different substances are themselves an example of convergent evolution. Rather, I am arguing that a category based on convergent evolution, i.e., one that includes both human and squid eyes, and a category “addiction” including addiction to all substances, stand in the same relation to categories that are genuine natural kinds. They are examples of categories that share a property cluster but not in virtue of a common mechanism.

## Addiction and Loss of Control

The DSM defines addiction based on patterns of use, that is to say, behavioral criteria. But experimental models focus on something like loss of control. Why? When seeking a mechanism to explain the property cluster of addiction – something in virtue of which addiction applies as a property to individuals – we need to look for a mechanism *for something*. In this section, I’ll discuss why loss of control is an appropriate candidate to be explained mechanistically. For it is the mechanisms that seek to explain loss of control with which I will take issue.

First, one cannot look for a mechanism responsible for a behavior on the neurocognitive level without a prior understanding of the psychological function that explains behavior: there is no brain area for aggression, rather, there are functions, realized neuroanatomically, that explain aggressive behaviors (18).

Second, a behavioral definition necessarily casts the net too widely, as it cannot distinguish between an addict and an enthusiast in the right conditions [Sinnott-Armstrong and Pickard (19), though, see Griffiths (66) for reasons why we might not want to make such a distinction]. By enthusiast I mean someone who has a strong desire for something but one that is completely within the agent’s control. Sinnott-Armstrong and Pickard give the example of an avid golfer, though in theory this could apply to an avid drug user who is not addicted (be it from not being addicted yet, not having the predisposition, etc.). An enthusiast who needs to travel somewhere for business where he or she cannot obtain drugs has good reason not to use drugs, and, will subsequently abstain. However, suppose for the purposes of this thought experiment that the enthusiast never encountered such a situation as a matter of chance: what, behaviorally, would distinguish him from an addict? Sinnott-Armstrong and Pickard (19) therefore suggest that having a motivational structure that includes a loss of control is an essential part of addiction. Of course, having no control but no desire to use drugs also does not constitute addiction, so it must be both^8^.

The HPC account need not entirely ignore behavior as a part of addiction. I suggest that the addictive phenotype, i.e., addiction-related behaviors as described in the DSM, are the surface properties, explained by the causally basic properties which can be loosely described at a coarse grain as the functions underlying having a strong and habitual desire and a loss of control.^9^ Further, it is the causally basic properties that are supposed to be definitive of the kind, and thus these properties constitute a valid definition, whereas the behaviors are not causally basic. Finally, person-level properties such as loss of control stand in just the right relation to subpersonal functional mechanisms: the latter can account for the occurrence of the former without fully (reductively) explaining it (20).

I emphasize that the causally basic properties can be described – in the sense that they can be grouped together – as “loss of control,” not that loss of control is itself a causally basic property. For loss of control, when understood at anything but the coarsest of grains, is far too heterogeneous to count as property, and thus a candidate to be causally basic^10^. First of all, loss of control itself is a broader category than loss of control in addiction, which refers to some specific sort (or sorts) of loss of control. Further, addiction theorists disagree on how to cash out loss of control in terms of the functions that generate behavior which can be described as not controlled (to some extent) by the agent.

Indeed, each theorist I discuss cashes out loss of control in a different way: for the aberrant learning hypothesis, the causally basic property is the habit-based behaviors that are not sensitive to executive control systems, whereas for the frontostriatal dysfunction model it is impaired activity in the executive systems themselves – understood at a fine grain to include multiple functional systems such as incentive salience attribution, emotion regulation, executive override functions, and others – that are the causally basic properties. Notably, some even propose different numbers of causally basic functions related to loss of control: the aberrant learning and incentive sensitization hypotheses give one functional neuroadaptation primacy^11^ whereas the frontostriatal model suggests the equal contribution of several functions.

When I say loss of control in general, I mean to refer to those causally basic properties hypothesized by the respective theories discussed here that can be grouped together as underlying some sort of loss of control, and not some property itself.

The key point is that the way in which addiction according to the disease paradigm putatively maps to HPC kinds can now be stated more precisely. The surface properties are addictive behaviors, the causally basic properties are the functions suggested by the theorists related to loss of control, and the mechanisms are the neurocognitive adaptations proposed to be causally relevant to those functions.

## Theories of Addiction

The disease conception of addiction is close to the received view in cognitive neuroscience. Melis and colleagues sum up the view well:

[N]ot different from traditional diseases, drug addiction bears with it a number of biological abnormalities. which justifies the label disease. Although repetitive use of drugs affects different organs. the primary target appears to be the brain – thus brain disease [Ref. (21), p. 102].

In this section, I’ll discuss some various proposals for relevant mechanisms. I’ll then detail how the suggested mechanisms in each theory fail to account for a class of substances.

Varieties of the disease conception suggest different mechanisms on the level of functional neurocircuits – that is, sets of brain areas with assigned functions – to explain the occurrence of the causally basic properties. I’ll discuss a few prevalent models: (1) Everitt and Robbins (7) suggest that aberrant associative learning results in uncontrollable habits realized in dopaminergic projections to and within the dorsal striatum, and that addiction involves transfer of behavioral control from voluntary reward-based systems to these nearly reflexive habit learning systems via dopaminergic projections from the ventral to the dorsal striatum. (2) Robinson and Berridge (22–24) suggest that the mesolimbic dopamine (DA) system malfunctions in addiction. They suggest that the function of the system is to compute the “incentive salience” of a stimulus, or how much an organism “wants” (as opposed to “likes”) that stimulus. They hypothesize that, in addiction, the system changes to habitually overvalue drug-related stimuli at the expense of other classes of stimuli. (3) Volkow and colleagues (6, 10, 25) suggest that several neurocircuits involving various prefrontal control regions underlie different aspects of loss of control in addiction, and that these circuits “conspire” to overwhelm cognitive control systems.

In each case, evidence shows that at least one substance of abuse violates a major prediction of the theory such that it is not obvious that the putative mechanism is operant in those cases. For the former two, cannabis is the exception; for the third, opiates are.

### Theory one: Aberrant learning and the dorsal striatum

Everitt, Robbins, and colleagues (7, 26) suggest that a dopaminergic neurocognitive circuit focused around the dorsal striatum plays a central role in the generation of addictive behaviors. Specifically, they suggest that behavioral control shifts from normal reward-based learning functions realized in the mesolimbic DA system^12^ to habit-based learning functions realized in the dorsal striatum.

The dorsal striatum is involved in unmediated stimulus-response associations, as opposed to mediated S-R associations (27). A mediated S-R association is one that is related to some other mental factor. The relationship between food (S) and eating (R) is generally a mediated response: a rat will eat food if it’s hungry, but not if it’s full (under normal circumstances). Thus the food-stimulus/eating-behavior association is mediated by hunger levels. These associations are realized in a neurocognitive circuit involving the ventral striatum, ventral tegmental area (VTA), and OFC – essentially, the mesolimbic DA system (27).

An unmediated association is an automatic and involuntary association between stimulus and response, such as walking into a dark room and reaching for/moving a light switch.

Everitt and Robbins suggest that addiction occurs when drug-taking and drug-seeking behavior cease to be generated by mediated S-R mechanisms and begin to be generated by unmediated ones. In functional terms, drug-taking and drug-seeking become automatic, involuntary responses to drug-related stimuli (or conditioned cues) in addiction, whereas in drug use that is not addiction, they are mediated by some psychological factor. In an elaborate series of experiments, Everitt and Robbins have demonstrated that activation of the dorsal striatum is involved in the animal model of relapse: drug-seeking behavior after prolonged abstinence in response to conditioned stimuli.

A major prediction of this model is that these dopaminergic circuits in the dorsal striatum change over time in response to the influx of dopamine from the drug of choice. This can be measured experimentally in a few ways. First, dopamine type 2/type 3 (D2/3) receptor availability will decrease in the dorsal striatum in addicted individuals. This lowered receptor availability is the brain’s response to repeated, externally induced dopaminergic innervations: rather than over-activating constantly, the brain raises the threshold for activation in terms of required D2/3 levels. To be clear, it is not the receptors themselves that are hypothesized to play a causal role in addiction, rather, measuring receptor levels is intended to indirectly measure the causally efficacious dopamine activity^13^. And this prediction is confirmed in many substances, including cocaine (28), alcohol (29), opiates (30), methamphetamine (31), and nicotine (32). Yet D2/3 receptor availability is not affected within statistically significant levels in either adolescents (33) or adults (34) recovering from cannabis addiction.

A second experimental model is to investigate the level of dopamine transporters in and around the dorsal striatum *in vivo*, which also decreases as a response to repeated, artificially induced influxes of dopamine. In a study by Leroy and colleagues (35), striatal and extrastriatal dopamine transporter levels were compared between three groups: healthy controls, individuals addicted to nicotine, and individuals addicted to nicotine and cannabis. The latter two groups showed no significant difference in dopamine transporter levels, but both demonstrated a significant difference compared to controls, suggesting that cannabis – unlike nicotine – does not decrease dopamine transporter levels in and around the dorsal striatum.

Therefore, the data available on dopamine activity in the dorsal striatum in cannabis addiction that has been collected thus far points to the conclusion that cannabis, in contrast to virtually every other drug of abuse, does not involve the neuroadaptation proposed to underlie addiction in this theory.

### Theory two: Incentive sensitization

Robinson and Berridge (22–24) agree with Everitt and Robbins that regular drug use starts out affecting the mesolimbic DA system, but disagree on the function of that system as well as the location of the salient neuroadaptations for the transition to addiction. They suggest the mesolimbic DA circuit computes incentive salience, or the extent to which an organism “wants” a stimulus, as opposed to – and independent of – the extent to which the organism “likes” it, and this function itself is compromised in addiction.

All drugs of abuse produce a rush of dopamine to the VTA, which transmits more throughout the circuit (21, 36). According to the incentive sensitization theory, this causes the system to improperly compute the incentive salience value of the drug in terms of its objective benefit, significantly overvaluing the substance that caused the DA release. Over time, the brain builds up a tolerance to higher DA levels, and as a result, salience values of stimuli across the board, so to speak, are devalued – except for drug-related stimuli, which are re-overvalued each time the drug is consumed. In functional terms, addiction occurs when we automatically value drug-related stimuli as the stimuli with the highest incentive salience value (whether we consciously endorse this valuation or not). This process is referred to as *incentive sensitization*.

A major prediction of this theory, then, is that non-drug-related rewards such as food, water, and sex (though not money, presumably because it becomes associated in the brains of addicts with obtaining drugs) generate less activation in mesolimbic DA system areas, especially the ventral striatum, than in conspecifics, and drug-related stimuli generate greater activation. And this prediction has been confirmed in addiction to alcohol (37), cocaine (38), and nicotine (39). But again, this prediction is not borne out with cannabis: heavy cannabis users exhibit greater ventral striatum activation in response to cannabis-related stimuli more than non-users, but they also exhibit greater activation to non-drug-related stimuli (40)!^14^ Further, Wolfing and colleagues (41) found that verbal reports as well as psychophysiological measurements (EEG and skin conductance resonance or SCR) showed that habitual cannabis users value valenced non-drug stimuli greater than do controls^15^.

An experimental paradigm related to incentive sensitization at the neural level is behavioral sensitization. In this paradigm, animal models are noted to initially have increased spontaneous locomotor activity in response to drugs of abuse, and this activity decreases with repeated exposure, hence behavioral sensitization. And the process is hypothesized to mimic sensitization at the neural level [Varvel et al. (42); though, see Ref. (43) for a challenge to this connection]. Varvel and colleagues performed an elaborate series of experiments on comparisons between behavioral sensitization to methamphetamine and to cannabis. In all iterations in which there was evidence for behavioral sensitization to methamphetamine, there was no evidence for behavioral sensitization to cannabis.

Functionally, then, this theory runs into a problem explaining the occurrence of strong, habitual desire with loss of control in cannabis addiction. If drugs are the only thing of value to an agent (in the agent’s unconscious valuation), then the agent will predictably keep taking drugs at the expense of other rewards. But, according to this data, cannabis use doesn’t necessarily make one want cannabis at the expense of other things, it makes one want everything more. Thus it is not clear why the agent would prefer the drug over other non-drug rewards. In other words, it might explain the agent’s becoming a hedonist in general, but not an addict to a particular substance.

### Theory three: The frontostriatal dysfunction model

Volkow and colleagues (9, 44) proposed a decade ago that prefrontal cortical areas, involved in executive control and inhibition (among other functions) in normal brain function also underlie the loss of control aspect of addiction. Specifically, the systems are compromised in terms of overall functional capacity, and, as a result, exhibit decreased activity via a variety of measures. In other words, an agent’s normal self-control systems fail to block drug-taking and drug-seeking behavior, resulting in use that is out of the agent’s control.

More recently (10, 25), they have expanded this model to discuss particular aspects of compromised inhibition which are each assigned to a specific neurocircuit involving distinct subregions of the PFC; in large part this move is simply a reflection of a better understanding of the heterogeneity of PFC function [Goldstein and Volkow (10); for a review of PFC areas and their respective functions, see Ref. (45)]. Many of these circuits involve projections between the PFC and ventral striatum, however, I focus on PFC related data here.

While the full details of this intricate model are beyond the scope of this discussion, a crucial point is that the various neurocircuits are postulated not to independently promote aspects of the addictive phenotype but rather to feed off of each other and “conspire” to produce the addictive phenotype (6). Thus, for addiction to be a natural kind on this account, the set of mechanisms must explain addiction across substances.

However, opiates cause a problem for predictions involving several of the proposed circuits. Indeed, there are myriad differences between opiate and either cocaine or stimulant addiction summarized by Badiani and colleagues (1). I will focus here on the data they discuss that is most relevant to the frontostriatal theory.

According to Volkow and colleagues (10, 25), the medial PFC as a whole is involved in several relevant neurocircuits, including one underlying the balancing of immediate gratification with the attainment of long-term goals, and this circuit’s function is compromised in addiction. Because of the decreased activity in the area, the theory predicts that spiny neuron density will increase in the mPFC (as a homeostatic response to lowered neurotransmitter levels in virtue of decreased activity). And this result has been confirmed in cocaine, methamphetamine, and cannabis (46, 47). However, animal models of heroin addiction show the opposite: decreased spiny neuron density in the mPFC in abstinent rats (46). Further evidence regarding differences in mPFC morphology come from measuring long-term potentiation (LTP) in GABAergic synapses in animal models *in vivo*. Repeated cocaine exposure suppresses LTP in the mPFC, and, subsequently, abstinence facilitated LTP reformation (48, 49). Abstinence after repeated exposure to heroin, however, had no significant effect on LTP (50).

Moreover, distinct subregions of the medial PFC, especially the ventromedial PFC (vmPFC), are implicated independently of the mPFC as a whole in the function of other circuits relevant to self control in the PFC. Specifically, the vmPFC is involved in stimulus valuation and salience attribution as well as emotion regulation (10).

Yet evidence suggests that these subregions are differentially involved in addiction to cocaine and opiates. For heroin, context-induced reinstatement can be attenuated by inhibiting the vmPFC (51). This is plausible given the theoretical interpretation of the circuits in which the vmPFC is involved. For it would seem to be that the reason relapse can be cue induced in the first place is because the associated cues are given such a high valuation or attributed salience. However, for cocaine, context-induced reinstatement can be attenuated when the dorsal, but not the ventral mPFC is inhibited (52). Not only is this result functionally difficult to explain since the dmPFC is not implicated in valuation and salience attribution, but it demonstrates that the involvement of PFC neuroadaptations in addiction cannot be assumed to be the same across substances.

## Implications for the Category of Addiction

At least according to the best neuroscientific theories of addiction available, there is no mechanism underlying loss of control in addiction across substances. I thus argue that, barring further evidence in the future to the contrary, addiction is not a natural kind. In this section, I’ll discuss what ought to be done to find natural kinds in addiction science. First, however, I want to address the scope of the claim I’m making. Specifically, I want to address the possibility of what finding further evidence in the future to the contrary might mean for this claim.

### Natural kinds and the status of addiction science

Because the HPC view is committed to the best current science, its claims are always working hypotheses, up for revision in light of future empirical evidence. This means that when I claim “addiction is not a natural kind,” I am making a statement tantamount to a working hypothesis, warranted by the state of science at this time. However, there are two ways the science could change such that the data would no longer support my contention, and I want to address both possibilities. First, a future theory of the mechanisms of addiction could be introduced that would meet the criteria I’ve discussed for natural kindhood, i.e., that would explain addiction across substances in terms of a common causal mechanism or set of mechanisms. Such a theory could be a reformulation or augmentation of a current theory, or a novel hypothesis. Second, the experimental findings that have warranted the theories already discussed may be invalidated. I discuss each of these possibilities in turn, and suggest reasons as to why I am still justified in making the claim that addiction is not a natural kind.

What if future research finds mechanisms of addiction that truly apply to addiction across substances? In that event, the basis for warranting kind claims would change to respect the new findings, and, subsequently my thesis would be unwarranted – barring research even further in the future to the contrary of *those* claims. However, such a possibility is in principle entirely consistent with making the claim at the current time that addiction is not a natural kind, since this sort of claim always entails a status as a working hypothesis.

Because mechanisms that unify kinds are what warrant the making of natural kind claims, and because these mechanistic findings are always themselves up for revision in light of new evidence, a kind theorist is left with two options: make no kind claims at all until the science is essentially static (e.g., it seems rather safe to assume that the unity of an element category in chemistry like gold will not be challenged), or make kind claims that are tantamount to working hypotheses. But it is the current generalizations that we make that enable science to progress: the claim that addiction is not a natural kind is based on past science, and aimed to help future science (and philosophy; see S 6). The empirical literature discussed here was generated because scientists were studying the nature of what they took to be a valid category, and this study has produced scientific progress. In other words, science is well served by making and augmenting working hypotheses about the unity/disunity of various categories.

A distinct possibility is that the theories that warrant my claims about the disunity of the category addiction themselves will be falsified by future science. And this possibility is at least *prima facie* plausible. For instance, Ahmed (53, 54) rightly questions the addiction status of animal models who have been given no choice but to repeatedly take the drug presented to them, which calls into question many of the experimental findings on which these theories are based. If a radical version of this claim were true such that none of the current theories proposed were valid enough to warrant any claims about natural kinds, what would that mean for my claim that addiction is not a natural kind? Samuels (55) suggests that when studying a putative category where no potential, reasonably supported mechanistic basis has been proposed, we take the working hypothesis that the category is a HPC kind based on similarity of surface properties. And addiction would seem, at least *prima facie*, to qualify as having similar surface properties (addiction-related behaviors) for instances of addiction across substances, though a more robust discussion of the behavioral and epidemiological patterns of addicts to various substances would need to happen before such a claim could be made with any amount of confidence.

However, I do not understand the possibility that the experimental basis for these theories may be invalidated to undermine my claim that addiction, as the science stands right now, is not a natural kind. For such an argument is a criticism of what is currently the dominant paradigm in addiction, and a distinct one from the point I am making. The dominant paradigm of addiction, as I understand it, suggests that: (1) results from the animal models of addiction used over the last half century or so project to instances of addiction in humans and (2) that mechanistic explanations of addiction to one substance project to instances of addiction to other substances^16^. Both of these claims are still part of the dominant paradigm; I am challenging the latter claim and arguments like Ahmed’s are challenging the former. And while I am sympathetic to Ahmed’s argument, I am not, in this discussion myself, challenging the projectability from animal models to humans. Rather, I am making a separate challenge to a dominant view that still largely accepts this tenet.

### The search for kind terms

When investigation of a category previously thought to be a natural kind fails to reveal a mechanism underlying the property cluster, the natural kind theorist is committed to one of two alternatives to rectify the situation: category revision and category replacement (14, 56). The commitment to category replacement or revision in light of empirical evidence is part and parcel of the HPC view’s commitment to scientific practice. A common mechanistic basis among members of a category is what warrants natural kind claims, and since the mechanistic claims are empirically revisable, so are the claims about which categories count as natural kinds.

To again paraphrase Griffiths (14), this is not to say that nothing is happening in individuals we refer to as addicted. It just means that classifying them as suffering from addiction might not be particularly illuminating as to what’s going on in their brains. And the strategies of category replacement and/or revision are the next step in the search for categories that will be illuminating.

In this section, I’ll go over how these two strategies might be used by an addiction scientist to find valid natural kinds. I shall also explain why I prefer the category replacement strategy, where we cease to discuss in terms of “addiction” simpliciter and come to discuss patients in terms of “S-addiction” or “T-addiction.”

### Category revision

Category revision means rethinking the extension of the category, i.e., the set of entities to which it refers. And there is a move available to the addiction theorist to salvage some category similar to addiction.

For whichever mechanistic account of addiction ends up being correct, addiction theorists might simply revise the extension of “addiction” to refer to cases of addiction caused by the right mechanisms. Thus, if the aberrant learning model is correct, it might be the case that there is a natural kind of disorder encompassing addiction to cocaine, nicotine, alcohol, etc., but not cannabis. When looking for the common mechanisms of addiction, researchers might just cease to demand that patients putatively addicted to cannabis display evidence of those specific neuroadaptations. (If the frontostriatal dysfunction account is correct, we’d have to jettison heroin addiction from the natural kind.)

### Category replacement

Another option is to replace addiction as a category with related categories that better track distinct mechanisms. Notably, the mechanisms underlying loss of control in addiction do not seem to vary across users of the same substance; minimally, I am not arguing here that they do. Thus, nothing I have suggested contradicts – and indeed, the evidence might be said to corroborate^17^ – the idea that “cocaine addiction” and “cannabis addiction” for example, are natural kinds.

Moreover, under such a conception it might be the case that addiction to two (or more) substances end up using the same set of mechanisms. For instance, suppose cocaine and methamphetamine are found to have the same mechanism or set of mechanisms that end up being the consensus for underlying loss of control in addiction. Then patients addicted to either substance would be members of a category that counted as a single natural kind.

### Reasons to prefer category replacement

While both category revision of addiction (to exclude cannabis or opiates) and category replacement of addiction with “S-addiction,” “T-addiction,” etc., are both equally valid strategies, I find the second option more attractive.

Whichever theory is correct, category replacement, at least utilizing the strategy I have suggested, might lead us to the same place as category revision. That is, it is possible that if the aberrant learning hypothesis is correct, and if this is the case, investigation would then determine that S-/T-addiction are the only two real kinds, where S is cannabis and T is all substances except cannabis, just as we would with category revision if the aberrant learning hypothesis were correct.

I argue that if this is the case, and we would arrive at the same conclusion either way, then the claim will be on far better theoretical standing if arrived at via the second strategy. For it would be reached as a conclusion of investigation rather than as a stipulated premise.

Fundamentally, the lesson I hope we would draw from the discrepancies between substances of addiction is that, given the current state of addiction science, assuming a unitary category of addiction is no longer justified. At the beginning stages of investigation – for instance, when it was discovered that all drugs of abuse target or act on the mesolimbic circuit with dopamine (36) – it might have been reasonable to infer as a working hypothesis the unity of addiction from the unity of acute use. But that was when our best evidence about long-term addiction was indirect, i.e., from short-term use, and the situation is now different.

One could object that perhaps addiction *per se* is analogous to a higher taxa category in species biology. That is, S-addiction is like the species human and addiction is, for example, the genus primates^18^. But this analogy does not work: in the case of taxa, the relevant mechanisms are evolutionary, and all taxa share some ancestry (indeed, biological categories only count as taxa if they do, otherwise the putative taxon is merely a homoplasy). However, in the case of addiction, the relevant mechanisms are physiological/neurocognitive, and these mechanisms either play a causally efficacious role in the drug-related behavior of addicts to a given substance, or they do not.

## Conclusion: Implications for Scientists and Philosophers

I hope I have demonstrated in this paper that natural kinds are just a simple yet relatively precise way to capture the conceptual commitments of theoretical constructs such as “addiction” (or “emotion,” or “species”).

In this concluding section, I wish to discuss why various members of the scientific and philosophical community might benefit from replacing “addiction” with “S-addiction.” All researchers make predictions or inferences on the basis of category membership. And in all cases, researchers benefit from using categories that are supported by underlying mechanisms.

### Basic science

Basic scientists are concerned with making the most powerful explanations they can, and the relevance of natural kinds is thus direct. Natural kinds are categories that are maximally predictive, and categories that fail to be natural kinds *ipso facto* fail to be maximally predictive, no matter how much their members have in common.

Natural kinds *just are* the categories scientists ought to use for the basis of making predictions, and thus for the design of experiments. It is telling that experimental cohorts are rarely if ever composed of patients with addictions to distinct substances (that is, a patient with S-addiction and one with T-addiction in the same cohort, as opposed to comorbidity studies): conclusions would be very difficult to draw.

### Clinical science

Clinicians are most concerned with what ameliorates the condition of their patients. But again, mechanisms matter. Supposing that, say, the aberrant learning hypothesis is correct, and we begin to develop drugs that target the dorsal striatum. Those drugs wouldn’t work for instances of cannabis addiction. Similarly, while behavioral measures might target higher-level phenomena seemingly independent of mechanism, the mechanism still matters. Therapies that work to create and reinforce new associations will work well for fixing maladaptive mediated associations but not unmediated ones (or, minimally, better for the former type than the latter). Thus if loss of control in addiction to various drugs of abuse is underwritten by distinct mechanisms, different therapies will be differentially effective.

Thus clinicians are best served by devising treatments for “S-addiction” and “T-addiction” rather than addiction *per se*^19^. Simply put, natural kinds form the best basis for diagnosis and treatment just as they do prediction and induction.

### Philosophy

Moral psychologists are interested in attributions of moral properties like responsibility and autonomy based on an individual’s mental composition (or psychology). The relationship between physical and moral properties – assuming that there is any – is a topic of great debate and by no means one I shall enter here.

Indeed, it is logically possible that the mechanisms underlying varieties of loss of control in addiction are irrelevant for the ascription of moral properties: it may be that the high level properties themselves are all that is needed. It might not be a knock against a naturalistic theory of moral psychology that the mechanistic level doesn’t matter for moral purposes. And if the mechanistic level doesn’t matter, then a category’s being a natural kind doesn’t matter for the purposes of generalizations to subsume moral ascriptions.

However, there is reason to think that it might. Pickard (5) suggests that addicts maintain a middle ground in ascriptions of moral autonomy between full responsibility and full blamelessness. Madva (under review) claims (primarily in regard to implicit bias) that having some but not full control over a behavior might be a good psychological basis on which to ascribe such partial responsibility. Indeed, he suggests, following Holton (57) that addicts might exhibit such partial control insofar as “raising the stakes,” i.e., the rewards for quitting, has significant effects on who is able to abstain. In other words, if someone can quit in some circumstances and not others, then that is a good indicator of partial control.

Might the mechanisms underlying loss of control in addiction have relevance to the magnitude of control lost? It is at least *prima facie* an hypothesis worthy of empirical investigation. If Everitt and Robbins are correct, then, devolution of behavioral control from ventral to dorsal striatum corresponds with a higher degree of loss of control than before the “switch.” And thus addictions not involving this morphological change would not entail the same magnitude of loss of control. Further, if Holton and colleagues are right that the ability to quit under a greater variety of circumstances indicates greater control, then it is also telling that cannabis users are statistically less likely to seek treatment than users of other substances (58).

In other words, it is at least a hypothesis worth investigating empirically (and a theory worth investigating conceptually) that different addictions, in virtue of different mechanisms, differentially contribute to the ascription of moral properties.

## Conflict of Interest Statement

The author declares that the research was conducted in the absence of any commercial or financial relationships that could be construed as a potential conflict of interest.

## Footnotes

^1^While there are many practical uses of defining addiction, including legal and personal reasons (19), I focus on the three mentioned above as I understand them to be intimately related: clinical diagnosis and treatment for mental disorders depends on the underlying neurocognitive structure of the patient. And while the relationship between what psychology tells us about the mind/brain and how we attribute moral status such as moral responsibility is doubtless a complex issue, rather, I am simply saying that realist moral psychology requires that the attribution of moral properties be in some way influenced by how the mind actually works.

^2^These features are often referred to as essences, though I will avoid this term as it is loaded in implying something metaphysically special, i.e., Aristotelian essences (12), and I wish to avoid such confusion. However, essences need not be metaphysically special.

^3^There are two kinds of kinds, so to speak: natural and social. Very roughly, members of natural kinds share properties in virtue of something natural or physical and members of social kinds share their properties in virtue of something social (11).

^4^I am not critiquing any definition of addiction in favor of any other, rather, I am pointing out inadequacies with various definitions. My main point is that whatever definition of addiction one chooses, there are problems applying it to all cases of what we call addiction. Against the disease conception, I argue that no mechanism can account for the properties of an agent understood as “addiction” in all cases; for the purposive conception I argue that the definition itself has trouble explaining some cases of what we call addiction.

^5^For instance, Hacking (59) endorses family resemblances but not clusters (where the former must count as somehow co-sufficient and the latter, as I have explained, need not) as well as restricts natural kinds to human-independent categories. Yet stricter still are more “traditional” accounts of natural kinds which pose essences as necessary and sufficient conditions for kindhood [e.g., Ref. (60)] On such accounts, addiction would not be a natural kind: it has no metaphysically special essence, and it is a kind defined at least in part by human behaviors.

^6^I am thankful to Bennett Foddy for bringing up this point in review.

^7^More precisely, the members must have those properties generated by mechanisms/sets of mechanisms that are tokens of the same type.

^8^Sinnott-Armstrong and Pickard (19) add a third criterion, that the addiction causes harm, though they also suggest that loss of control might be inherently harmful. This part of the definition is not necessary for the purposes of this discussion; my arguments do not turn on any explanation of harm.

^9^I should note that not all theorists endorse some form of loss of control as central [e.g., Ref. (61)]. However, the theorists discussed, that is, those in the disease paradigm of addiction, do. See my (62) for a discussion on the unity of addiction in Pickard’s theory.

^10^I am thankful to Bennett Foddy for raising this point in review.

^11^Everitt and Robbins (7) do discuss the role of the prefrontal cortex distinct from the dorsal striatum, however, they also later suggest that prefrontal neuroadaptations may temporally precede addiction (63).

^12^The postulated function of the mesolimbic DA system differs between theories of addiction. For Everitt and Robbins, it is involved in the learning of reward value, whereas, for, e.g., Robinson and Berridge (22, 23), it is involved in the attribution of incentive salience.

^13^I am thankful to Serge Ahmed for raising this point in review.

^14^The findings of Nestor and colleagues suggest cannabis is a problem for *any* theory of addiction that posits greater VS activation in response to drug-related stimuli in the face of decreased activation to non-drug-related “rewarding” stimuli. An influential theory of addiction that I have not discussed makes a similar prediction with a similar neural basis but with a different functional story. The “allostatic” theory of addiction [e.g., Ref. (64)] suggests that repeated, externally induced dopaminergic innervations and the resulting hypodopaminergic state generate an anhedonic affective/nocioceptive state in addicted individuals, who then use the drug of their preference to retain normal hedonic functioning. Similarly, addiction is supposed to result in reward-system driven overvaluation of drug-related and undervaluation of non-drug-related stimuli, except here the over/undervaluations are computed in terms of reward value rather than incentive salience. But the suggested physiological basis for this computation is still the mesolimbic DA system, and thus the evidence from cannabis is problematic.

^15^However, by this study’s measurements – in contrast to the Gruber data – habitual cannabis users did not overvalue non-drug rewards to the same extent as they did drug rewards in comparison to controls.

^16^I understand projection in this sense to roughly mean an inference from a pattern in a specific subset of samples to another, larger specific set or subset (65). See Boyd (11) for further discussion of projectability and natural kinds.

^17^I should note that this might be a relic of the experimental construct which tests a group of individuals in treatment for addiction to a particular substance against a control group of non-users or occasional users of the same drug.

^18^I am thankful to Alex Madva for raising this point in response to an earlier draft.

^19^This is a distinct point from the fact that addiction treatment must be individually tailored, although that point is certainly valid. However, even the greatest amount of variation in treatment for any disorder will contain some matter of commonality, and which categories we base those generalizations on will matter. In other words, person-to-person variation in treatment for addiction is still variation on the same theme. Combining my argument with the personal variation insight suggests that there should be person-to-person variation in “S-addiction” as well as “T-addiction” but that the addiction-related properties of individuals in group S are statistically far likelier to resemble each other more than any given individual in group T.
